# High prevalence of *ALPK3* premature terminating variants in Korean hypertrophic cardiomyopathy patients

**DOI:** 10.3389/fcvm.2024.1424551

**Published:** 2024-07-05

**Authors:** Seung Woo Ryu, Won Chan Jeong, Geu Ru Hong, Jung Sun Cho, Soo Yong Lee, Hyungseop Kim, Jeong Yoon Jang, Sun Hwa Lee, Dae-Hwan Bae, Jae Yeong Cho, Ji Hee Kim, Kyung-Hee Kim, Jang Won Son, Beomman Han, Go Hun Seo, Hane Lee

**Affiliations:** ^1^ Research and Development Center, 3billion, Inc., Seoul, Republic of Korea; ^2^ Division of Cardiology, Yonsei University College of Medicine Severance Hospital, Seoul, Republic of Korea; ^3^ Division of Cardiology, Daejeon St. Mary’s Hospital, College of Medicine, The Catholic University of Korea, Daejeon, Republic of Korea; ^4^ Division of Cardiology, Department of Internal Medicine and Research Institute for Convergence of Biomedical Science and Technology, Pusan National University Yangsan Hospital, Pusan National University School of Medicine, Yangsan, Republic of Korea; ^5^ Division of Cardiology, Department of Internal Medicine, Keimyung University Dongsan Medical Center, Daegu, Republic of Korea; ^6^ Division of Cardiology, Department of Internal Medicine, Gyeongsang National University School of Medicine, Gyeongsang National University Changwon Hospital, Changwon, Republic of Korea; ^7^ Division of Cardiology, Department of Internal Medicine, Jeonbuk National University Medical School, Jeonbuk National University Hospital, Jeonju, Republic of Korea; ^8^ Division of Cardiology, Department of Internal Medicine, Chungbuk National University Hospital, Cheongju, Republic of Korea; ^9^ Department of Cardiovascular Medicine, Chonnam National University Hospital, Chonnam National University Medical School, Gwangju, Republic of Korea; ^10^ Division of Cardiology, Department of Internal Medicine, College of Medicine, The Catholic University of Korea, Seoul, Republic of Korea; ^11^ Division of Cardiology, Incheon Sejong Hospital, Incheon, Republic of Korea; ^12^ Division of Cardiology, Department of Internal Medicine, Yeungnam University Hospital, Daegu, Republic of Korea

**Keywords:** *ALPK3*, premature terminating variant, hypertrophic cardiomyopathy, whole exome sequencing, Korean HCMP population

## Abstract

**Background:**

The alpha-protein kinase 3 (*ALPK3*) gene (OMIM: 617608) is associated with autosomal recessive familial hypertrophic cardiomyopathy-27 (CMH27, OMIM: 618052). Recently, several studies have shown that monoallelic premature terminating variants (PTVs) in *ALPK3* are associated with adult-onset autosomal dominant hypertrophic cardiomyopathy (HCMP). However, these studies were performed on patient cohorts mainly from European Caucasian backgrounds.

**Methods:**

To determine if this finding is replicated in the Korean HCMP cohort, we evaluated 2,366 Korean patients with non-syndromic HCMP using exome sequencing and compared the cohort dataset with three independent population databases.

**Results:**

We observed that monoallelic PTVs in *ALPK3* were also significantly enriched in Korean patients with HCMP with an odds ratio score of 10–21.

**Conclusions:**

We suggest that *ALPK3* PTV carriers be considered a risk group for developing HCMP and be monitored for cardiomyopathies.

## Introduction

Hypertrophic cardiomyopathy (HCMP) affects approximately 1 in 500 to 1 in 200 people worldwide (1–3). It is characterized by the thickening of myocardial ventricular walls and is both phenotypically and genetically heterogeneous. While it is commonly inherited in an autosomal dominant fashion within families with incomplete penetrance and variable expressivity, there are also autosomal recessive forms, and non-Mendelian forms may exist (4). To date, there are 51 genes described to be associated with HCMP (1, 5–9). Identifying the genetic basis is important for more precise clinical management and family testing, as clinical prognosis and the possibility of intervention can vary depending on the underlying cause (9–12). With the advent of next-generation sequencing (NGS), it has become readily accessible to sequence many genes simultaneously as panels or exome/genome sequencing. The diagnostic yield from various genetic testing averages around 30%, suggesting that there are still more monogenic gene–disease associations and/or complex non-Mendelian forms yet to be discovered (3, 5, 13).

While most genes associated with HCMP are sarcomere-related genes such as *MYBPC3* and *MYH7*, few non-sarcomere-related genes have been identified (14). One of these non-sarcomere-related genes is the *ALPK3* gene coding for alpha-protein kinase 3 (ALPK3), which is a 1,705 amino acid long atypical protein kinase predicted to play an essential role as a transcription factor during cardiomyocyte differentiation (15–17). Biallelic premature terminating variants (PTVs), resulting in loss of function in *ALPK3*, are associated with autosomal recessive, familial hypertrophic cardiomyopathy 27 (CMH27, OMIM: 618052) characterized by early-onset severe dilated cardiomyopathy in infants that progresses to HCMP over time (18). Studies using mouse models have shown that *ALPK3* is expressed early in cardiogenesis and remains expressed in cardiomyocytes (16). *ALPK3*-null (−/−) mice develop both ventricular hypertrophy and dilation suggesting the underlying pathomechanism of the disease is loss of function of the *ALPK3* gene (19). However, despite the available evidence, the relationship and pathogenic mechanism between *ALPK3* and HCMP remain unclear.

Recent studies have suggested autosomal dominant inheritance of *ALPK3*-associated HCMP. The original study by Almomani et al. (18) observed that family members of patients with biallelic pathogenic *ALPK3* were at risk of developing later-onset HCMP. In their study, they identified 2 out of 10 family members of autosomal recessive patients who were heterozygous carriers and presented with HCMP, suggesting a possible risk association (18). A subsequent study from Herkert et al. (20) reported that monoallelic rare *ALPK3* variants accounted for approximately 2.5% of the unexplained HCMP in 1,548 Dutch patients and 10% of US patients with unexplained HCMP. Lopes et al. (21) reported that heterozygous *ALPK3* PTV carriers were enriched in their HCMP cohort. They discovered that 12 out of 770 HCMP patients in their discovery cohorts and 32 out of 2,047 HCMP patients in the validation cohort had monoallelic *ALPK3* PTVs such as non-sense, frameshift, and canonical splice site variants. They reported an odds ratio (OR) of 16 when compared to presumed healthy controls in gnomAD v3.1.2 (21). Dai et al. (22) demonstrated that 4 out of 793 HCM cases of East Asians have monoallelic PTVs in *ALPK3* with an odds ratio of 5.72 compared to the gnomAD v2.1.1 control.

These previous studies were predominantly performed on European Caucasian descent, except for one study performed on the Chinese population. Here, using a large East Asian HCMP cohort, we sought to replicate the previous studies by assessing the genetic burden of *ALPK3* PTVs in Korean HCMP patients. A total of 2,366 Korean patients with non-syndromic HCMP were compared to matched controls. Heterozygous PTVs in *ALPK3* were significantly enriched with an odds ratio between 10 and 21, which is consistent with previous studies.

## Methods

### Participants

The HCMP patient cohort comprised 2,366 Korean patients who underwent exome sequencing at a single reference laboratory in South Korea between 2019 and 2023. The inclusion criteria for the cohort included exhibiting left ventricular hypertrophy (LVH) as a primary phenotype and being predicted as an East Asian from the genetic data. Patients who did not meet these criteria were excluded. Clinical evaluations were conducted by clinicians from 16 different hospitals and clinics across the country. Since the study was conducted in a diagnostic setting and all the samples and data were de-identified throughout, Institutional Review Board (IRB) approval was not required.

### Exome sequencing and bioinformatic analysis

Exome sequencing was performed using high molecular weight genomic DNA extracted from whole blood samples using QIAamp DNA Blood Mini Kit (Qiagen) or buccal swab samples using AccuBuccal DNA Preparation Kit (Accugene). Exome capture was performed using xGen Exome Research Panel v2 (Integrated DNA Technologies, Coralville, IA, USA), and sequencing was performed using the NovaSeq 6000 platform (Illumina, San Diego, CA, USA) as 150 bp paired-end reads. Alignment to the GRCh37 human reference genome was performed using BWA-MEM2 (v2.2.1), and SAMtools v1.18 was used for bam file sorting and marking duplicates (23, 24). Recalibration and variant calling for single nucleotide variants (SNVs) and small insertion/deletion variants (indels) were performed using GATK v4.2.14. Copy number variants (CNVs) were identified using CoNIFER v0.2.2 and 3bCNV, an internally developed tool that uses depth-of-coverage information of each exon (25, 26). High-quality variants (allele depth ≥ 20, variant allele fraction ≥ 0.25 for heterozygous variants) were annotated, filtered, and classified using EVIDENCE v4, which incorporates Ensembl Variant Effect Predictor (VEP) for annotation and the American College of Medical Genetics and Genomics (ACMG) guideline for variant classification (27–29). The splicing prediction was performed using SpliceAI and SpliceVi (https://splicevi.io/), a publicly available in-house developed SpliceAI visualization tool (30). The filtered and classified variants were manually reviewed by medical geneticists and physicians. The variants that can most likely explain the patient's phenotype were selected for reporting.

## Results

### Patient demographic

The age distribution of the Korean HCMP patients was 2–99 years old, with a median age of 61 years (mean 59 years). Sixty-six percent were male patients (1,564/2,366). Pathogenic variants in the cardiovascular disorder genes were identified in 20.4% of patients (482/2,366). In 18 of these patients, more than one variant/gene was reported as likely causal for the cardiovascular phenotype. As in a previous study (6), *MYBPC3* and *MYH7* variants accounted for the majority of findings, each accounting for 40.7% (196/482) and 24.9% (120/482) of the total diagnoses, respectively. The third most frequently identified gene *TNNI3* accounted for 12.2% of the total diagnoses (59/482).

### *ALPK3* premature terminating variants identified in HCMP patients

**Figure 1:**
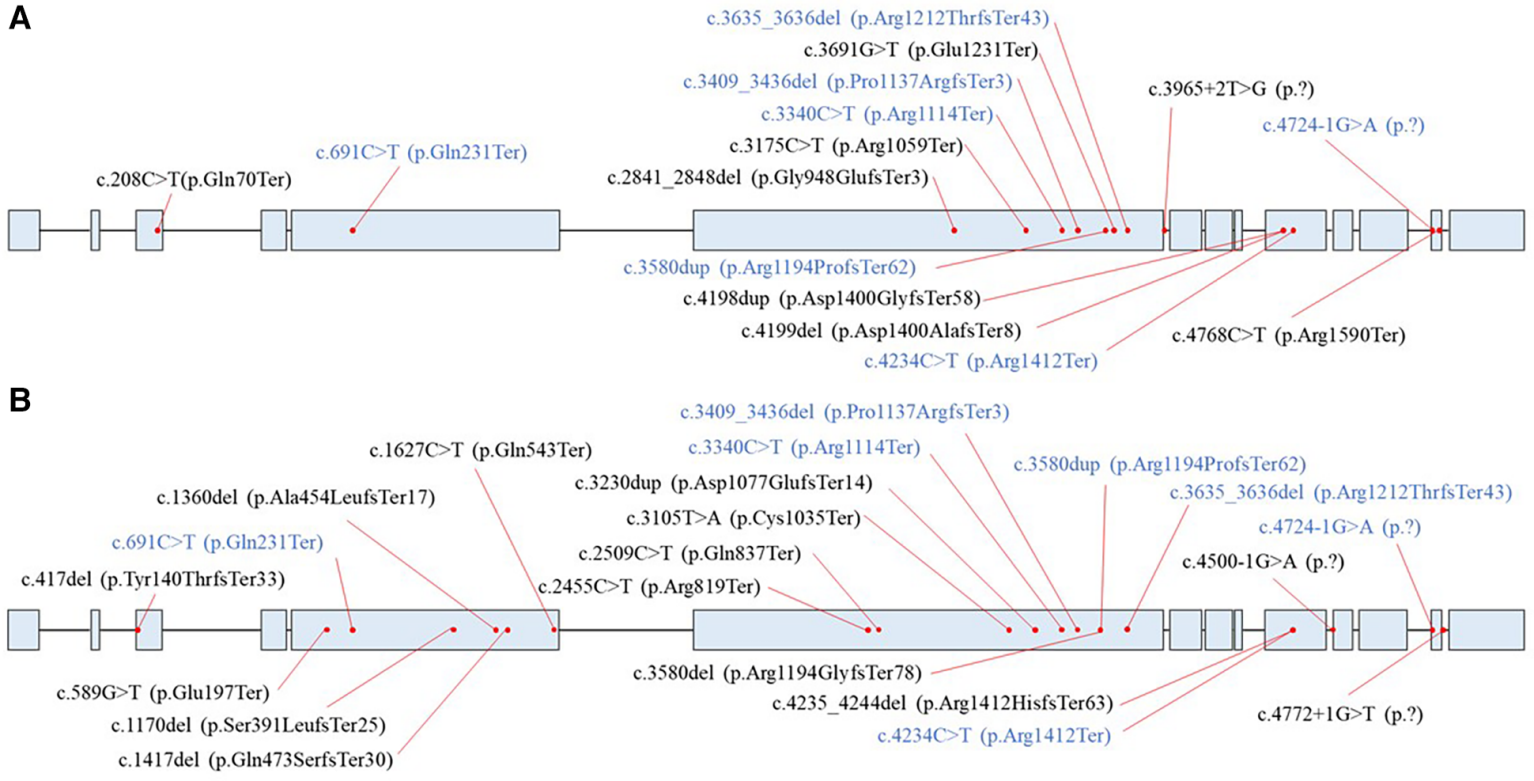
Distribution of *ALPK3* (NM_020778.5) premature terminating variants. (**A**) Variants identified in the HCMP patient cohort. (**B**) Variants identified in the control databases. Blue represents variants that were present in both the HCMP cohort and control data sets.

A total of 36 unrelated HCMP patients out of 2,366 patients (1.5%) carried rare pathogenic or likely pathogenic (P/LP) PTVs in *ALPK3*, classified based on the ACMG guidelines (Figure 1A, Table 1). The median age of these patients was 52 years, with a mean of 53 (+/- 17.1) years. The youngest patient was 17 years old. All patients exhibited left ventricular hypertrophy, which is a hallmark of HCMP. Arrhythmia and syncope were the second most common phenotypes (Table 1). With 7 of the variants recurring, a total of 14 unique variants were identified: 7 non-sense (stop gain) variants, 5 frameshift variants, and 2 splicing variants located in the essential canonical splice site. *In silico* splicing prediction algorithm, SpliceAI predicted the consequence of the two splicing variants, namely, NM_020778.5:c.3965 + 2T>G (p.?) and NM_020778.5:c.4724-1G>A (p.?), to introduce a new stop codon (Supplementary Figures S1A,B). Of the 14 unique variants, 6 variants were novel variants that have not been described in any database or literature to our knowledge (Table 2). None of the HCMP patients had a copy number variant (CNV) spanning at least three consecutive exons in *ALPK3*. For one patient, the variant was inherited from an affected parent, who was tested later by exome sequencing and therefore not included in the 36 patients, consistent with an autosomal dominant inheritance pattern.

**Table 1:** List of *ALPK3* PTV carrier HCMP patients. Phenotype information provided for the exome sequencing order is shown. NM_020778.5 transcript is used for the cDNA position. LVH, left ventricular hypertrophy.

Patient ID	HGVSC (NM_020778.5)	HGVSP (NP_065829.4)	Age (year)	Sex	Primary phenotype	Additional phenotype
Patient 1	c.208C>T	p.Gln70Ter	58	Male	LVH	
Patient 2	c.691C>T	p.Gln231Ter	78	Male	LVH	
Patient 3	c.691C>T	p.Gln231Ter	53	Male	LVH	
Patient 4	c.691C>T	p.Gln231Ter	66	Male	LVH	
Patient 5	c.691C>T	p.Gln231Ter	26	Male	LVH	
Patient 6	c.691C>T	p.Gln231Ter	70	Male	LVH	Syncope, pain
Patient 7	c.2841_2848del	p.Gly948GlufsTer3	64	Male	LVH	
Patient 8	c.3175C>T	p.Arg1059Ter	65	Male	LVH	
Patient 9	c.3340C>T	p.Arg1114Ter	24	Male	LVH	Left ventricular diastolic dysfunction
Patient 10	c.3409_3436del	p.Pro1137ArgfsTer3	32	Male	LVH	Arrhythmias
Patient 11	c.3409_3436del	p.Pro1137ArgfsTer3	34	Male	LVH	Proteinuria
Patient 13	c.3409_3436del	p.Pro1137ArgfsTer3	31	Male	LVH	
Patient 14	c.3409_3436del	p.Pro1137ArgfsTer3	63	Female	LVH	Arrhythmias
Patient 15	c.3580dup	p.Arg1194ProfsTer62	47	Male	LVH	
Patient 16	c.3580dup	p.Arg1194ProfsTer62	51	Male	LVH	Hypertension
Patient 17	c.3580dup	p.Arg1194ProfsTer62	40	Male	LVH	Heart failure
Patient 18	c.3635_3636del	p.Arg1212ThrfsTer43	51	Male	LVH	Hypertension
Patient 19	c.3635_3636del	p.Arg1212ThrfsTer43	70	Male	LVH	
Patient 20	c.3691G>T	p.Glu1231Ter	43	Female	LVH	Arrhythmia, nerve conduction abnormalities
Patient 21	c.3965+2T>G	p.?	61	Male	LVH	
Patient 22	c.4198dup	p.Asp1400GlyfsTer58	63	Male	LVH	
Patient 23	c.4199del	p.Asp1400AlafsTer8	77	Male	LVH	Proteinuria
Patient 24	c.4234C>T	p.Arg1412Ter	50	Male	LVH	Heart failure
Patient 25	c.4234C>T	p.Arg1412Ter	51	Male	LVH	Pain
Patient 27	c.4234C>T	p.Arg1412Ter	82	Male	LVH	
Patient 28	c.4234C>T	p.Arg1412Ter	41	Male	LVH	
Patient 29	c.4234C>T	p.Arg1412Ter	59	Male	LVH	
Patient 30	c.4724-1G>A	p.?	55	Male	LVH	Syncope
Patient 31	c.4724-1G>A	p.?	58	Female	LVH	
Patient 32	c.4724-1G>A	p.?	48	Male	LVH	
Patient 33	c.4724-1G>A	p.?	67	Male	LVH	Heart failure
Patient 34	c.4724-1G>A	p.?	51	Male	LVH	Syncope
Patient 35	c.4724-1G>A	p.?	39	Male	LVH	
Patient 36	c.4724-1G>A	p.?	87	Female	LVH	
Patient 37	c.4768C>T	p.Arg1590Ter	28	Male	LVH	
Patient 38	c.4768C>T	p.Arg1590Ter	17	Male	LVH	Syncope

**Table 2:** *ALPK3* variants and their consequences identified in HCMP patients.

Variant position (GRCh 37)	HGVSc (NM_020778.5)	HGVSp (NP_065829.4)	Variant consequence	Number of patients	Previous reported	ClinVar	ACMG
15-85370740-C-T	c.208C>T	p.Gln70Ter	Stop gain	1	This study	-	Likely pathogenic
15-85383201-C-T	c.691C>T	p.Gln231Ter	Stop gain	5	This study	-	Likely pathogenic
15-85400805-ACCCCAGGT-A	c.2841_2848del	p.Gly948GlufsTer3	Frameshift	1	This study	-	Likely pathogenic
15-85401144-C-T	c.3175C>T	p.Arg1059Ter	Stop gain	1	Almomani et al. (2016), Lopes et al.(2021), van Velzen et al. (2018)	VCV000488984.18	Pathogenic
15-85401309-C-T	c.3340C>T	p.Arg1114Ter	Stop gain	1	–	VCV001414127.4	Pathogenic
15-85401376-AGCCCTCCCAAGAGGA GAAGTTCCCAGGG-A	c.3409_3436del	p.Pro1137ArgfsTer3	Frameshift	4	Dai et al. (2022)	-	Pathogenic
15-85401543-T-TC	c.3580dup	p.Arg1194ProfsTer62	Frameshift	3	–	VCV000636490.6	Pathogenic
15-85401599-GGA-G	c.3635_3636del	p.Arg1212ThrfsTer43	Frameshift	2	This study	-	Likely pathogenic
15-85401660-G-T	c.3691G>T	p.Glu1231Ter	Stop gain	1	This study	-	Likely pathogenic
15-85402623-T-G	c.3965+2T>G	p.?	Canonical splice site	1	This study	-	Likely pathogenic
15-85405928-T-TG	c.4198dup	p.Asp1400GlyfsTer58	Frameshift	1	Dai et al. (2022)	-	Pathogenic
15-85405970-C-T	c.4234C>T	p.Arg1412Ter	Stop gain	6	Dai et al. (2022)	VCV000636381.9	Pathogenic
15-85410547-G-A	c.4724-1G>A	p.?	Canonical splice site	7	–	VCV001498045.5	Pathogenic
15-85410592-C-T	c.4768C>T	p.Arg1590Ter	Stop gain	2	Lopes et al. (2021)	VCV000620214.3	Pathogenic

Almomani et al. (18), Lopes et al. (21), van Velzen et al. (31), Dai et al. (22).

### Clinical features of *ALPK3* premature terminating variant carriers

Of the 36 unrelated individuals carrying a PTV in *ALPK3*, additional clinical features were collected for 24 individuals (Table 3). For the 18 patients with the age-of-onset information available, the mean age of onset was 42 years old (SD, 16.3) for 18 individuals. The average max left ventricular thickness was 19 mm (SD, 5.3) with an average left ventricular ejection fraction (LVEF) of 64.1% (SD, 11.4). Of the 24 patients, 11 patients had atypical left ventricular morphology, 8 were asymmetric, and 5 were concentric. Seven patients had experienced heart failure.

**Table 3:** Clinical LVH features of *ALPK3* variant carriers.

Patient ID	Follow-up duration (yr)	ECG	LVH morphology	Max wall thickness (mm)	Left ventricular ejection fraction (LVEF%)
Patient 1	2	LVH(+)	Concentric	13	70
Patient 2	11	LVH (+), RBBB	Asymmetric	28	69
Patient 3	14	LVH(+)	Concentric	19.2	64
Patient 5	<1	LVH(+)	Asymmetric	28	67
Patient 6	2	LVH(+)	Atypical	20	67
Patient 7	-	LVH(+)	Atypical	21	63
Patient 11	2	LVH(+)	Atypical	28.6	58
Patient 12	2	LVH(+)	Concentric	12	56
Patient 13	-	LVH(+)	Atypical	14	69
Patient 14	3	LVH(+)	Atypical	15	58
Patient 15	7	LVH(+)	Asymmetric	21	81
Patient 16	<1	LVH(+)	Asymmetric	23	64
Patient 18	2	LVH(+)	Asymmetric	26	59
Patient 21	12	LVH(-)	Atypical	20	20.3
Patient 22	2	LVH(+)	Atypical	19	57
Patient 24	2	LVH(+)	Atypical	27	60
Patient 25	3	LVH(+)	Asymmetric	19	74
Patient 26	6	LVH(+)	Atypical	15	71
Patient 27	5	LVH(+)	Asymmetric	19.3	72
Patient 30	10	LVH(+)	Atypical	20	65
Patient 33	6	LVH(+)	Asymmetric	27	79
Patient 34	16	LVH(+)	Atypical	14	64
Patient 35	8	LVH(+)	Concentric	13.6	70
Patient 36	1	LVH(-)	Concentric	16	62

### *ALPK3* premature terminating variant carriers are enriched in HCMP patients

We investigated if *ALPK3* PTVs are enriched in our Korean HCMP patient cohort by comparing our HCMP cohort with three independent control cohorts as follows.

#### 22,448 East Asian population from gnomAD v4.0

**Figure 2:**
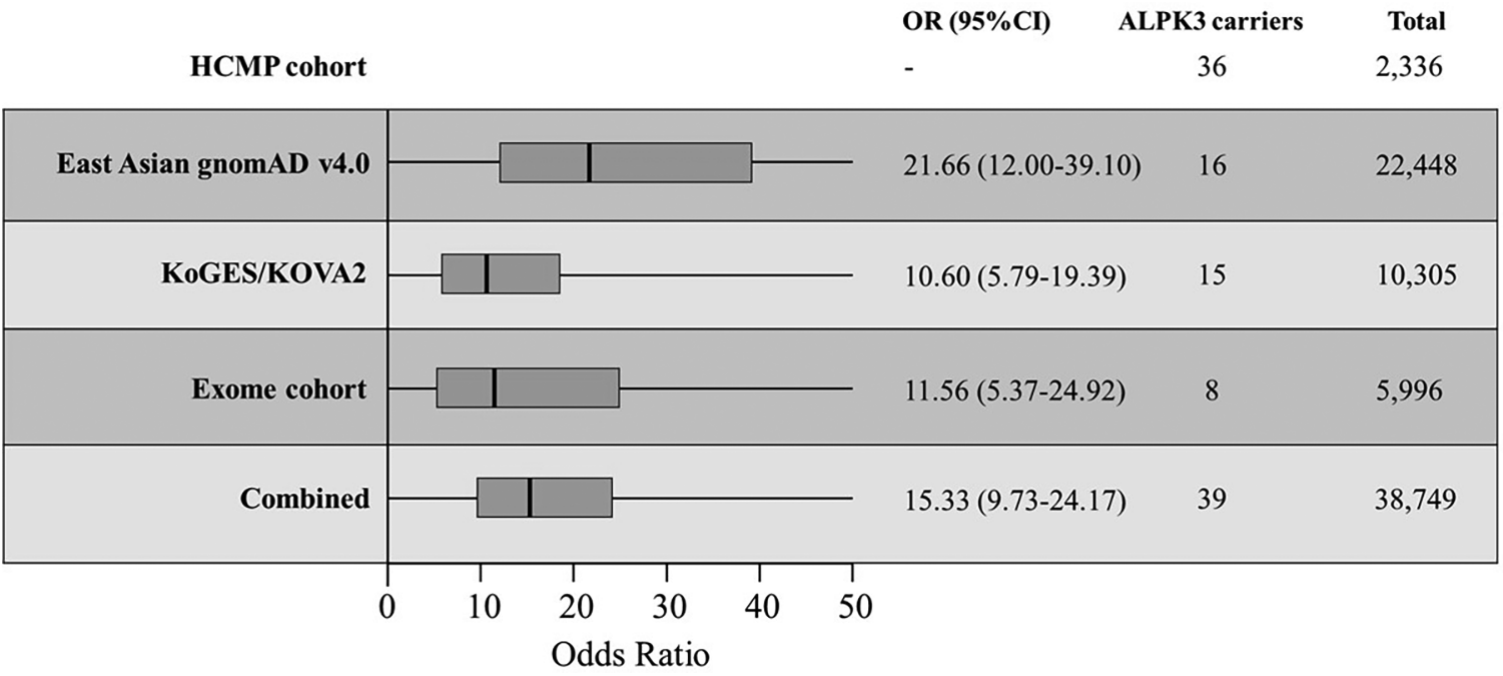
The odds ratios (OR) of different comparisons. Korean HCMP cohort consisted of 2,336 patients and were compared against the East Asian gnomAD v4.0 database, combined KoGES and KOVA2 database, internal exome database, and all combined. The thick vertical line is the calculated OR with the box representing the 95% CI.

The first comparison was made to the 22,448 East Asian population dataset within the Genome Aggregation Database (gnomAD v4.0, https://gnomad.broadinstitute.org/), the largest publicly available control database providing 807,162 exomes/genome sequencing data from the general population assumed to be healthy (32, 33). There were 14 unique *ALPK3* PTVs across 16 individuals out of 22,448 individuals (0.07%) (Table 4). All 14 variants were extremely rare with minor allele frequency (MAF) of <0.1% in the East Asian population. All 16 variants were heterozygous and classified as likely pathogenic or pathogenic based on the same classification algorithm used for classifying variants identified in our cohort. Eight variants were frameshift, five non-sense, and one essential splice site. SpliceAI predicted the consequence of the splicing variant c.4772+1G>T (p.?) to introduce a new stop codon (Supplementary Figure S1C). The odds ratio (OR) of this comparison was 21.66 (36/2,330 vs. 16/22,432; 95% CI: 12.00–39.10) (Figure 2).

**Table 4:** *ALPK3* variants identified in control cohorts.

HGVSc (NM_020778.5)	HGVSp (NP_065829.4)	HCMP cohort (*N*)	gnomAD v4.0 East Asian control cohort (*N*)	KoGES control cohort (*N*)	KOVA2 control cohort (*N*)	Exome control cohort (*N*)
c.208C>T	p.Gln70Ter	1	–	–	–	–
c.417del	p.Tyr140ThrfsTer33	–	2	–	–	–
c.589G>T	p.Glu197Ter	–	1	–	–	–
c.691C>T	p.Gln231Ter	5	2	2	2	3
c.1170del	p.Ser391LeufsTer25	–	–	–	1	–
c.1360del	p.Ala454LeufsTer17	–	1	–	–	–
c.1417del	p.Gln473SerfsTer30	–	1	–	–	–
c.1627C>T	p.Gln543Ter	–	1	–	–	–
c.2455C>T	p.Arg819Ter	–	–	1	–	–
c.2509C>T	p.Gln837Ter	–	1	–	–	–
c.2841_2848del	p.Gly948GlufsTer3	1	–	–	–	–
c.3105T>A	p.Cys1035Ter	–	1	–	–	–
c.3175C>T	p.Arg1059Ter	1	–	–	–	–
c.3230dup	p.Asp1077GlufsTer14		–	–	1	–
c.3340C>T	p.Arg1114Ter	1	–	–	1	–
c.3409_3436del	p.Pro1137ArgfsTer3	4	1	1	1	1
c.3580del	p.Arg1194GlyfsTer78	–	1	–	–	–
c.3580dup	p.Arg1194ProfsTer62	3	1	–	–	
c.3635_3636del	p.Arg1212ThrfsTer43	2	1	1	–	1
c.3691G>T	p.Glu1231Ter	1	–	–	–	–
c.3965+2T>G	p.?	1	–	–	–	–
c.4198dup	p.Asp1400GlyfsTer58	1	–	–	–	–
c.4235_4244del	p.Arg1412HisfsTer63	–	1	–	–	–
c.4234C>T	p.Arg1412Ter	6	–	–	3	1
c.4500-1G>A	p.?	–	–	–	1	
c.4724-1G>A	p.?	7	–	–	–	2
c.4768C>T	p.Arg1590Ter	2	–	–	–	–
c.4772+1G>T	p.?	–	1	–	–	–

#### 10,305 Korean individuals from KoGES and KOVA2

The second control dataset used for comparison was combined genomic data obtained through the Korean Genome and Epidemiology Study (KoGES) consisting of 5,000 genome sequencing data from assumed healthy Korean individuals and the Korean Variant Archive (KOVA2) consisting of 3,409 exome sequencing data and 1,896 genome sequencing data from assumed healthy Korean individuals (34, 35). The age distribution of the KoGES samples was 40–69 years, and 2,336 (46.7%) were male (34). Demographic information was not available for the KOVA2 dataset except for all healthy individuals.

In the KoGES database, four unique *ALPK3* PTVs were observed from five individuals. In the KOVA2 database, seven unique variants from 10 individuals were identified (Table 3). Together, 15 individuals carried *ALPK3* PTVs out of 10,305 individuals (0.15%). Together, four non-sense variants, four frameshift variants, and one essential splice site variant were observed from 15 individuals. SpliceAI predicted the consequence of the splicing variant, c.4500-1G>A (p.?), to introduce a new stop codon (Supplementary Figure S1D). All variants were observed as heterozygous. The odds ratio of this comparison to the combined cohort of KoGES and KOVA2 was 10.60 (36/2,330 vs. 15/10,290; 95% CI: 5.79–19.39) (Figure 2).

### Internal cohort of 5,996 patients

The third comparison was made to the internal dataset of 5,996 exome sequencing data from Korean patients collected between 2017 and 2023. Unlike the previous two control cohorts used for comparison, the internal exome sequencing data consisted of patients with suspected rare disorders but without HCMP phenotype. These patients had an age distribution between 1 year and 90 years with a median age of 22 years (mean, 28 years), and 3,280 were male (54%). There were 8 out of 5,996 patients (0.13%) with no indication of HCMP or cardiac phenotype identified with five unique heterozygous *ALPK3* PTVs (Table 3). The age distribution of these eight patients was between 1 year and 44 years with a median age of 9 years (mean, 14.6 years). Two variants were non-sense, two were frameshift, and one was an essential splice site variant predicted to result in premature termination. All five variants were also observed in the HCMP cohort. The odds ratio (OR) of this comparison was 11.56 (36/2,330 vs. 8/5,988; 95% CI: 5.37–24.92) (Figure 2).

### Combined cohort of 38,710 individuals

Finally, the comparison was made to the combined cohort of East Asian gnomAD v4.0 data, KoGES, KOVA2, and the internal dataset. Assuming no overlap between different databases, the combined cohort consisted of 38,749 individuals with 39 individuals (0.10%) carrying 21 different *ALPK3* PTVs (Figure 1B). The distribution of *ALPK3* PTV variants identified in the HCMP patient cohort and the patient control cohort was not significantly different (Figure 1). The final odds ratio was 15.34 (36/2,330 vs. 39/38,710; 95% CI:9.73–24.17) (Figure 2).

## Discussion

HCMP is one of the most commonly inherited cardiovascular diseases with a reported prevalence of 1 in 500 to 1 in 200 people globally and 1 in 300 people in South Korea (3, 36). While it contributes significantly to overall mortality and morbidity worldwide, early intervention and proper management can mediate sudden cardiac death and heart failure (37). Knowing the molecular basis of the disorder can help patients receive more precise interventions. Genetic testing is an efficient way to provide a molecular diagnosis to patients, prospective patients, and/or their family members at risk. That is why many (42%) of the 81 genes on the ACMG recommended secondary finding gene list are for cardiovascular diseases (10). However, despite over 50 different genes having been identified to be associated with HCMP, molecular diagnosis is only established for 30% of cases, suggesting that additional genetic loci and disease mechanisms for known genes remain to be discovered (1, 5, 8).

*ALPK3*, located on chromosome 15q25.2, is a protein kinase that is associated with autosomal recessive early-onset hypertrophic cardiomyopathy (OMIM: 618052) (15–18). This study replicates the findings that carriers of monoallelic *ALPK3* PTVs have a higher risk of developing late-onset HCMP. Out of 2,366 unrelated individuals with HCMP patients studied, heterozygous *ALPK3* PTVs were identified in 1.5% of total HCMP cases. Our study is the largest cohort studied so far and has also demonstrated comparable odds ratios of 15.33, which is similar to the odds ratio of 16.11 reported by Lopes et al. Although our cohort was limited to patients from South Korea, previous studies from other ethnic groups, including Turkey, the Dutch, the United States, the United Kingdom, and China, have all demonstrated a risk of acquiring HCMP with an *ALPK3* PTVs (13, 16–18). Thus, HCMP-associated risk for *ALPK3* does not seem to be limited to specific ethnic groups.

Although the first symptoms of HCMP can present as early as adolescence, the penetrance of HCMP is in general age-dependent with the average age of diagnosis being 41 years (20, 38–40). A study estimated the penetrance of HCMP by *ALPK3* heterozygous PTVs to be >95% by the age of 75 years for males and 80% for females (21). In our exome cohort, of the eight patients with *ALPK3* PTVs without clinical manifestation of HCMP, six individuals were below the age of 17. If these six individuals are excluded as they could potentially present with HCMP at a later age, the odds ratio becomes 25.07 (36/2,319 vs. 2/3,383; 95% CI:6.02–104.34), higher than the original calculation and higher compared to previous studies that did not account for the age of the control samples: 16.11 from Lopes et al. and 5.72–8.14 from Dai et al. (21, 22). However, because of the small number of cases, the 95% CI is wide, and further studies are needed with larger case numbers.

In our study, one patient (Patient 21), who is 63 years old, also carried a pathogenic variant in *MYBPC3* (NM_000256.3:c.2067+1G>A), which segregated with HCMP in the family. The *ALPK3* PTV did not segregate with HCMP in the family. There was no obvious difference in phenotype or severity between this patient and other family members. However, more dual-diagnosis cases would be needed to investigate if *ALPK3* heterozygous PTV modifies the phenotype.

Two studies by Herkert et al. and Dai et al. reported potential associations between HCMP and monoallelic deleterious *ALPK3* missense variants (20, 22). Although pathogenic assessment of missense variants is difficult and is often limited to *in silico* prediction, missense variants were suggested to represent a significant proportion of *ALPK3*-associated HCMP in both studies. In a study by Herkert et al. (20), 26 out of 36 identified variants in the Dutch population, and 6 out of 15 variants were missense in the US population were missense. Dai et al. (22) presented odds ratios of 3.17–3.61 for *ALPK3* missense variants. Our HCMP cohort did not have patients with a deleterious missense variant in *ALPK3* predicted by *in silico* tools REVEL (>0.6) or 3Cnet (>0.6). Further studies would be needed to define which missense variants are deleterious in *ALPK3* and determine their contribution to unexplained HCMP.

Long-term follow-up for HCMP patients with monoallelic *ALPK3* PTVs has suggested that the clinical course is more similar to patients with pathogenic variants in one of the sarcomere genes than to patients without (21). Given that patients with pathogenic variants in the sarcomere genes often have a more severe clinical prognosis than patients without, *ALPK3* PTV carriers are expected to have a relatively more severe clinical prognosis and therefore should be monitored for proper clinical management. Interestingly, a previous study found that 20% of the *ALPK3* PTV carriers had a subclinical increase in serum creatine kinases which is a feature associated with skeletal myopathy (21). Because our *ALPK3* PTV-positive patients have not been followed up for a long term yet or have been tested for serum creatine kinase level, further studies are needed to see if similar findings are observed.

In conclusion, we replicated previous studies suggesting that monoallelic PTVs in *ALPK3* increase the risk for late-onset HCMP. Although the odds ratio of 15.33 is lower than the odds ratio of *MYBPC3* PTVs, which are known to have odds ratios of >100, we believe that *ALPK3* should also be considered for inclusion in the secondary finding gene list and diagnostic laboratories should consider reporting monoallelic *ALPK3* PTVs when the patient is suspected of having a non-syndromic HCMP phenotype; negative carriers of *ALPK3* PTVs should be routinely monitored (10, 41). Although the disease mechanism is different, *MYH7* missense variants and *TNNI3* missense variants have similar OR as *ALPK3* with odds ratios of 14.4 and 14.3, respectively (41).

## Data Availability

The datasets presented in this study can be found in online repositories. The names of the repository/repositories and accession number(s) can be found in the article/**Supplementary Material**.
