# Supplementary material for: High prevalence of ALPK3 premature terminating variants in Korean hypertrophic cardiomyopathy patients
Source: Front Cardiovasc Med. 2024 Jul 5;11:1424551. doi: 10.3389/fcvm.2024.1424551 (PMC11259124; doi:10.3389/fcvm.2024.1424551)
Supplement: Supplementary file 1 [file Datasheet1.docx]

Supplementary Material

**
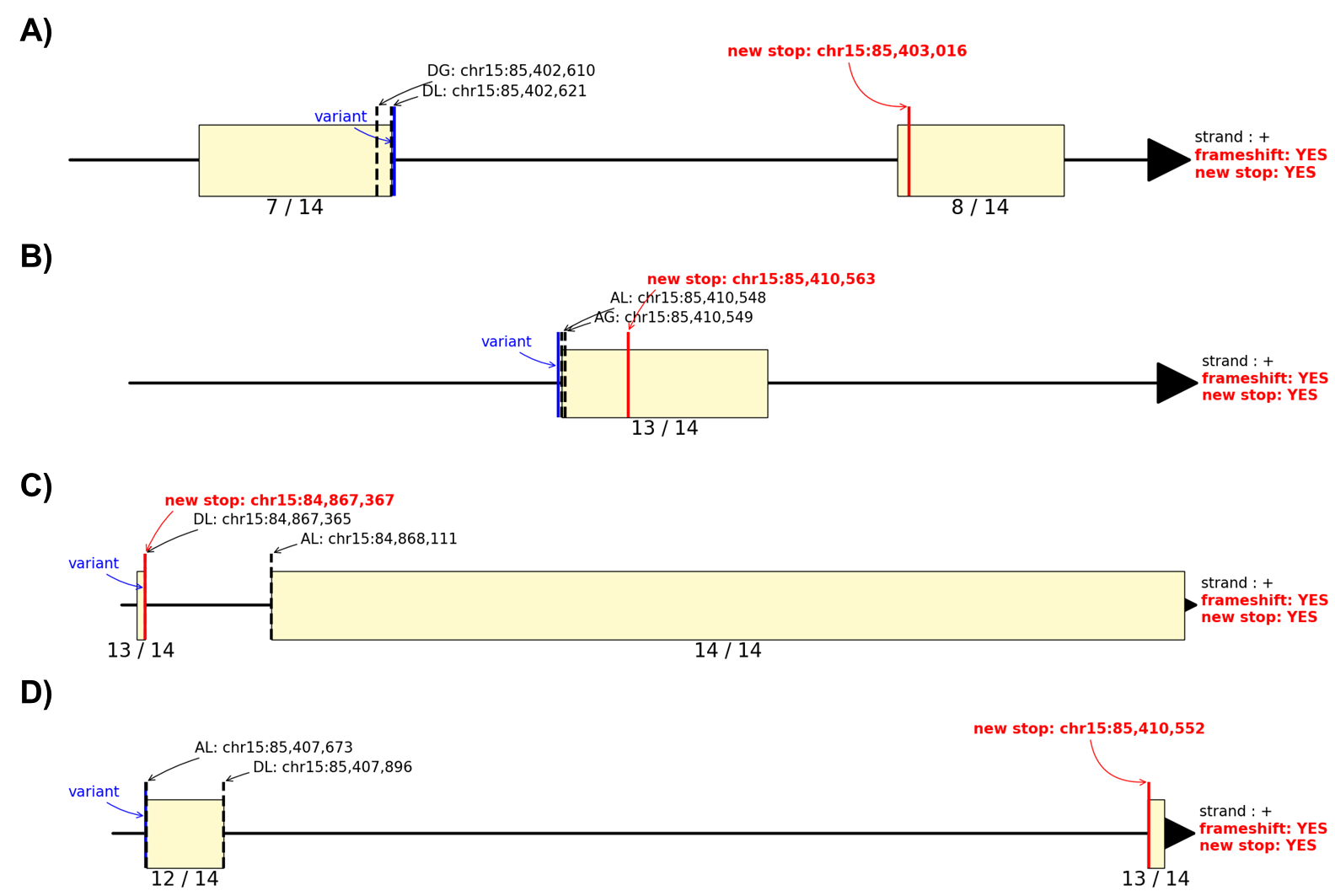
**

**Supplementary Figure 1.** Splicing prediction from SpliceAI* visualized using SpliceVi (https://splicevi.io/). A) Predicted consequence of NM_020778.5:c.3965+2T>G variant. AG: 0 (0 bp), AL: 0.41 (-141 bp), DG: 0.63 (-13 bp), DL: 0.98 (-2 bp). B) Predicted consequence of NM_020778.5:c.4724-1G>A variant. AG: 0.97 (2 bp), AL: 1.00 (1 bp), DG: 0 (0 bp), DL: 0.03 (49 bp). C) Predicted consequence of NM_020778.5:c.4772+1G>T variant. AG: 0 (0 bp), AL: 0.59 (745 bp), DG: 0.26 (-2 bp), DL: 0.99 (-1 bp). D) Predicted consequence of NM_020778.5:c.4724-1G>A variant. AG: 0.60 (2 bp), AL: 0.96 (1 bp), DG: 0 (0 bp), DL: 0.13 (224 bp).

*Raw Δscore and a max distance of 10,000 were used. Two highest computed scores were used for new stop prediction. AG = Acceptor gain, AL = Accepter loss, DG = Donor gain, DL = Donor loss.
